# MXene-Based Wearable Contact Lenses: Integrating Smart Technology into Vision Care

**DOI:** 10.1007/s40820-025-01863-5

**Published:** 2025-08-05

**Authors:** Arezoo Khosravi, Atefeh Zarepour, Ali Zarrabi, Siavash Iravani

**Affiliations:** 1 Department of Genetics and Bioengineering, Faculty of Engineering and Natural Sciences, Istanbul Okan University, 34959 Istanbul, Türkiye; 2 Department of Biology, Faculty of Arts and Sciences, Kocaeli University, 41001 İzmit, Kocaeli Türkiye; 3 Department of Biomedical Engineering, Faculty of Engineering and Natural Sciences, Istinye University, 34396 Istanbul, Türkiye; 4 Department of Research Analytics, Saveetha Dental College and Hospitals, Saveetha Institute of Medical and Technical Sciences, Saveetha University, Chennai, 600077 India; 5 Independent Researcher, W Nazar ST, Boostan Ave, Isfahan, Iran

**Keywords:** MXenes, Wearable contact lenses, Intraocular lenses, Drug delivery, Wearable electronics

## Abstract

MXene-based smart contact lenses demonstrate a cutting-edge advancement in wearable ophthalmic technology, combining real-time biosensing, therapeutic capabilities, and user comfort in a single platform. These devices take the advantage of the exceptional electrical conductivity, mechanical flexibility, and biocompatibility of two-dimensional MXenes to enable noninvasive, tear-based monitoring of key physiological markers such as intraocular pressure and glucose levels. Recent developments focus on the integration of transparent MXene films into the conventional lens materials, allowing multifunctional performance including photothermal therapy, antimicrobial and anti-inflammation protection, and dehydration resistance. These innovations offer promising strategies for ocular disease management and eye protection. In addition to their multifunctionality, improvements in MXene synthesis and device engineering have enhanced the stability, transparency, and wearability of these lenses. Despite these advances, challenges remain in long-term biostability, scalable production, and integration with wireless communication systems. This review summarizes the current progress, key challenges, and future directions of MXene-based smart contact lenses, highlighting their transformative potential in next-generation digital healthcare and ophthalmic care.

## Highlights

- MXene-based smart contact lenses seamlessly combine real-time biosensing, therapeutic functions, and enhanced user comfort, revolutionizing ocular health monitoring and treatment.
- The use of transparent MXene films enables features like photothermal therapy, antimicrobial protection, and dehydration resistance, significantly improving eye protection and disease management.
- While stability, scalability, and wireless integration pose hurdles, ongoing advancements suggest these lenses hold tremendous potential for transforming digital healthcare and ophthalmic care.

## Graphical abstract

**Figure:**
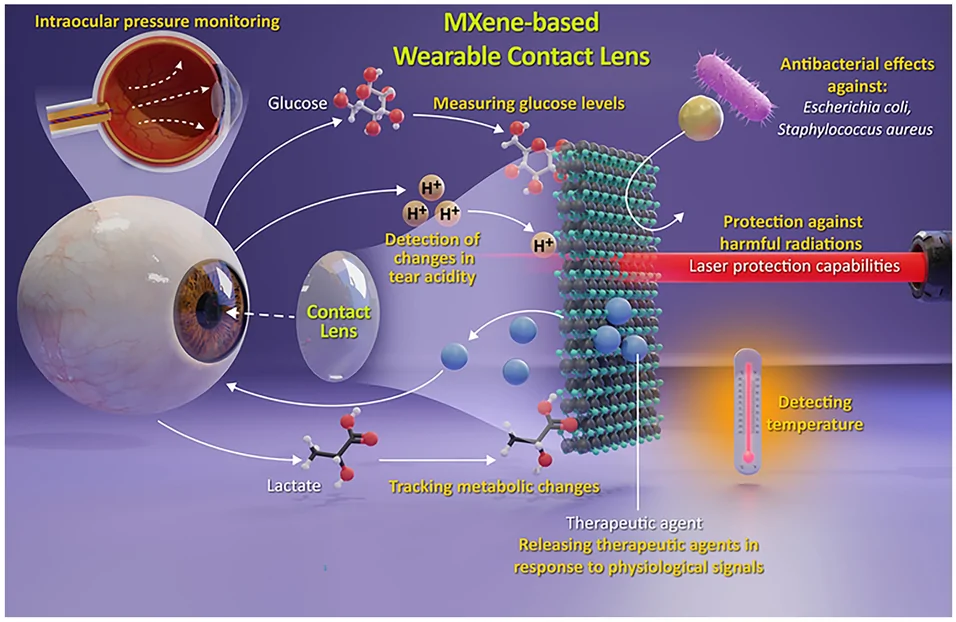

## Introduction

Contact lens biosensors are innovative devices that integrate various sensing technologies to monitor health parameters non-invasively through the eye [1, 2]. Wearable contact lens biosensors have emerged as a groundbreaking technology in the field of healthcare [3, 4]. These innovative devices, worn directly on the eye, provide a non-invasive and continuous monitoring method for different physiological parameters. With the integration of biosensors, these smart contact lenses (SCLs) can present real-time data on vital signs, glucose levels, and even detect markers of diseases [5–8]. Some of the key types of contact lens biosensors include glucose biosensors, which measure glucose levels in tears for diabetes management; pH sensors that detect changes in tear acidity to identify inflammation or infections; and lactate biosensors for tracking metabolic changes during physical activity. Additionally, drug delivery biosensors can release therapeutic agents in response to physiological signals, while bacterial infection sensors alert users to potential infections. Furthermore, biomarker sensors can detect specific health-related markers, and multi-parameter sensors combine several functionalities for comprehensive monitoring. Together, these biosensors offer valuable insights into ocular and systemic health, enhancing preventive care and management strategies [1, 9, 10].

The potential applications of wearable contact lens biosensors are vast [6]. For instance, in the field of diabetes management, these lenses can monitor glucose levels in tears, eliminating the need for frequent finger pricks [11, 12]. By wirelessly transmitting data to a smartphone or wearable device, individuals can track their glucose levels and make informed decisions about their insulin dosage or dietary choices [13]. However, current optical glucose sensors often involve complicated and lengthy fabrication methods, which hinder their practicality. Furthermore, wearable contact lens biosensors can play a crucial role in the early detection of diseases, but the readouts from these sensors are not suitable for accurate quantitative analyses, limiting their effectiveness in clinical applications [13]. By continuously monitoring biomarkers present in tears, these lenses can detect subtle changes that may indicate the presence of various health conditions. This early detection allows for prompt medical intervention, potentially improving patient outcomes and reducing healthcare costs [13, 14].

The design of wearable contact lens biosensors involves integrating miniature sensors and microelectronics onto a thin, flexible substrate that conforms to the shape of the eye. This technological feat enables a comfortable and unobtrusive wearing experience for users. Additionally, improvements in materials science have led to the production of biocompatible coatings that ensure long-term ocular health and reduce the potential for side effects [15]. However, like any emerging technology, wearable contact lens biosensors face challenges and considerations. Ensuring the accuracy and reliability of the biosensor measurements is crucial for their widespread adoption. Additionally, privacy and data security concerns must be addressed to safeguard the sensitive health information collected by these devices. Notably, some important challenges such as multifunctionality, self-power property, resistance to repetitive wear, flexibility, optical transparency, mechanical stability, and biocompatibility still need comprehensive evaluations [5]. The integration of nanomaterials in smart wearable contact lenses is transforming vision correction and health monitoring. These advanced materials enable the development of multifunctional lenses equipped with nanosensors that can monitor vital health parameters. Additionally, nanostructures enhance comfort by improving moisture retention and oxygen permeability, reducing dryness. Adaptive lenses that adjust tint based on light conditions offer protection from harmful UV rays, while embedded displays and sensors pave the way for augmented reality applications, allowing users to receive notifications and health alerts directly in their field of vision [16].

**Fig. 1:**
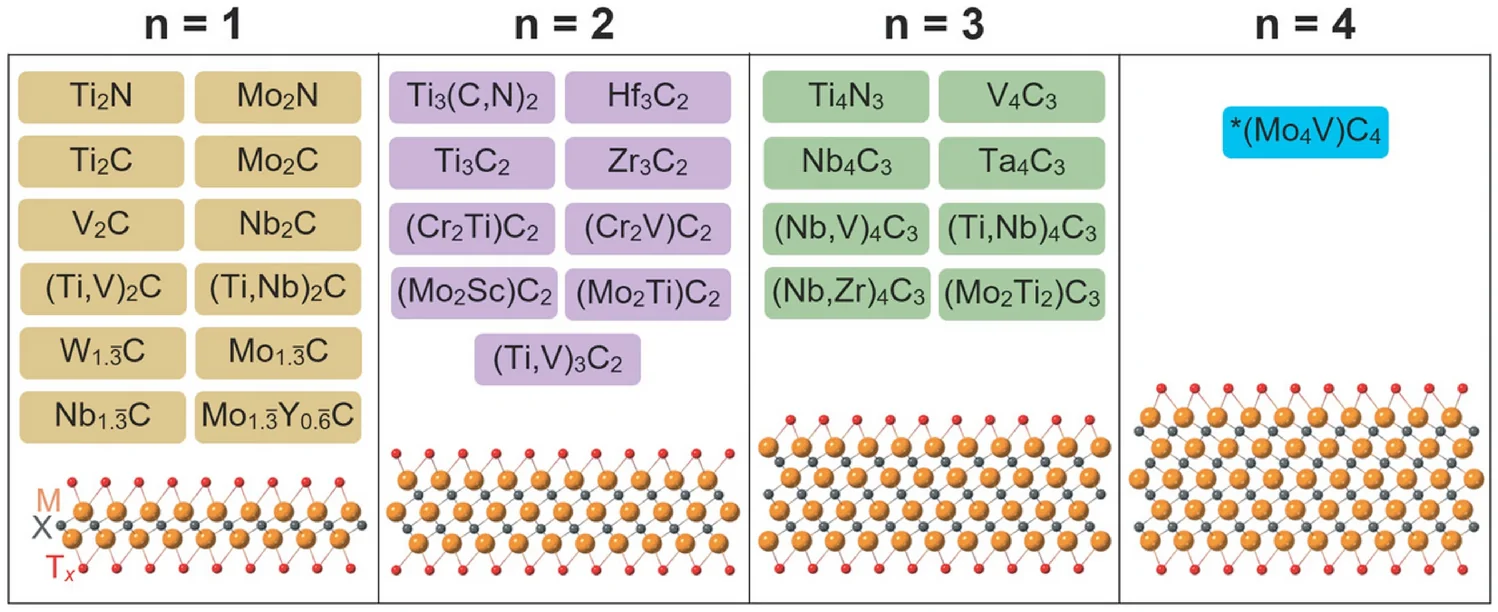
Some of the important MXene compositions, including M_2_X MXenes (n = 1), M_3_X_2_ (n = 2), and M_4_X_3_ (n = 3). Additionally, numerous types of MXene compositions have been investigated theoretically, and various solid-solutions have also been developed. Reproduced with permission from Ref. [28]. Copyright 2019 American Chemical Society

Intraocular lenses (IOLs) have become essential for restoring vision in cataract patients. However, current IOLs fall short when it comes to replicating the eye’s natural ability to accommodate. The remarkable optoelectronic properties of MXenes present a promising avenue for advancements in accommodating IOLs [17]. These materials boast high electrical conductivity, good optical transparency, hydrophilicity, flexibility, and biocompatibility, making them suitable candidates for this application. MXenes are two-dimensional (2D) materials consisting of transition metal carbides, nitrides, or carbonitrides with unique properties that make them attractive for incorporation into contact lens technology (Fig. 1). One of the key advantages of MXenes is their excellent electronic and electrochemical properties [18, 19]. This property allows for the integration of electronic components, such as sensors or microelectronics [20], directly into the contact lenses. By embedding MXenes within the lens structure, it becomes possible to create SCLs with built-in biosensors for real-time monitoring of various physiological parameters [21]. Additionally, MXenes exhibit exceptional mechanical properties, such as high strength and flexibility [22, 23], which are crucial for contact lens applications. These properties ensure that the lenses remain durable and comfortable for the wearer, even with the inclusion of additional components. Notably, MXenes have a high surface area, which can be utilized for drug delivery purposes [24–26]. Contact lenses incorporating MXenes can potentially serve as a platform for controlled release of medications, allowing for targeted and sustained drug delivery directly to the eye. This feature holds promise for the management of ocular diseases and conditions, providing optimized treatment options [27].

It is important to note that the addition of MXenes into the structure of contact lenses is still in the early stages of research and development. Challenges and considerations need to be addressed before their practical implementation. These include ensuring biocompatibility, minimizing potential adverse reactions, and optimizing the fabrication processes to ensure consistent and reliable performance. Research efforts are focused on exploring various MXene formulations, optimizing their properties, and understanding their interactions with the ocular environment. Indeed, the interaction of MXenes and their composites with biological systems presents a fascinating frontier in ocular health. Recent research reveals their remarkable biocompatibility [29–31]. This characteristic positions MXenes as promising candidates for targeted drug delivery systems [25, 32]. Moreover, their potential extends to biosensors specially designed to detect ocular diseases [33]. Diving deeper, scientists are exploring the electrochemical behavior of these materials. This exploration could pave the way for advanced sensing technologies for monitoring ocular conditions. Another noteworthy aspect is the versatility of MXenes [34]. Thus, they can be engineered to possess specific properties tailored for ocular applications. This adaptability opens avenues for creating more effective and targeted therapies. However, in addition to oxidative stability and scalability for commercial applications, comprehensive biocompatibility studies are necessary to confirm the safety and long-term effects of MXene-based contact lenses on ocular health.

This review aims to illuminate the remarkable advancements and innovations surrounding MXene-based wearable contact lenses. By exploring the latest research and developments, it seeks to provide a comprehensive overview of the multifaceted applications of MXenes and their composites in ocular technology, especially wearable contact lens biosensors. Furthermore, the review will explore how these cutting-edge materials enhance the performance of contact lenses, focusing on their unique properties, such as conductivity, flexibility, and biocompatibility. In doing so, it intends to highlight the potential for MXene integration in smart lenses, which could develop vision correction and health monitoring.

## Wearable Smart Contact Lenses (SCLs), from Advanced Biosensing and Monitoring to Therapy

Currently, the majority of contact lenses are designed with a restricted capacity, often limited to detecting a single biomarker in the eye. This includes important compounds such as glucose, lactic acid, potassium (K^+^), or calcium ions (Ca^2+^). While these lenses contribute valuable information, their singular focus limits their overall utility in comprehensive health monitoring. A major advancement in contact lens design would be the creation of lenses with enhanced capabilities with the ability of real-time monitoring of multiple chemical components, simultaneously. Such advancement could vastly enhance their potential as biomedical tools, providing a more holistic view of ocular health [5, 35, 36]. SCLs have emerged as a groundbreaking innovation, showcasing remarkable potential for non-invasive monitoring biophysical and biochemical signals. Biophysical signals related to eye health encompass both physical and electrophysiological indicators. Physical signals, such as temperature and intraocular pressure (IOP), play a vital role in assessing ocular health. Furthermore, electrophysiological signals, including electroretinography (ERG), provide valuable insights into retinal functionality. Smart contact lenses are valued for their ability to efficiently convert physical signals into user-friendly outputs, including electrical, optical, and microfluidic responses. In addition to physical signals, electrophysiological signals like ERG are essential for ophthalmic diagnostics. They play a crucial role in assessing retinal functionality. Monitoring variations in corneal surface potential enables effective evaluation of neuronal and non-neuronal cell responses within the retina under light stimulation. Employing advanced sensing materials in SCLs not only enhances the accuracy of monitoring but also opens new avenues for personalized healthcare solutions [35, 37].

SCLs possess advanced sensing components capable of tracking vital physiological signals within the eyes [35, 38]. For instance, IOP and ocular fluid composition, which includes crucial elements like glucose, cortisol, and various metabolites, can be effectively monitored. This makes SCLs not just a tool for vision correction, but an essential platform for health diagnostics. Recent developments have predominantly concentrated on SCLs with electrical signal outputs. These typically incorporate transparent electrode materials, biosensors, and wireless communication technologies. Designing SCLs that can track glucose levels while also assessing lactic acid or electrolyte concentrations all at once could open new way for improved management of conditions like diabetes and electrolyte imbalances. Moreover, the integration of electrical sensors within contact lenses could significantly enhance their capabilities. These sensors would not only facilitate the monitoring of biophysical signals like temperature and pressure but also enable the regulation of electroretinography. This means the lenses could actively monitor retinal responses to light stimulation or even stimulate visual neurons electrically. Such innovations could transform how ocular health is monitored and managed, ultimately leading to better patient outcomes and enhanced quality of life. However, challenges arise due to complicated fabrication processes and issues related to electromagnetic radiation. These factors significantly impede their potential for large-scale commercial adoption. In contrast, SCLs utilizing microfluidic and optical signal outputs present a promising alternative. They operate without the need for external power sources, making them more versatile [35, 39].

Wearable SCLs represent a groundbreaking intersection of optics, materials science, and healthcare technology, offering innovative solutions for real-time health monitoring and therapeutic interventions. These advanced devices leverage cutting-edge materials, such as MXenes, to provide enhanced functionalities that extend beyond traditional vision correction. One of the most significant advantages of wearable SCLs is their ability to facilitate continuous health monitoring. By integrating sensors capable of detecting various physiological parameters—such as intraocular pressure, glucose levels, or hydration status—these lenses can provide critical insights into a user’s health in real time. For instance, diabetic patients could benefit immensely from lenses that monitor glucose levels non-invasively, offering a more comfortable and less invasive alternative to finger-prick blood tests. However, the effectiveness of such monitoring systems relies heavily on the accuracy and reliability of the embedded sensors. Future research must focus on improving the sensitivity and specificity of these sensors to ensure that they can reliably detect fluctuations in health metrics. Furthermore, the challenge of miniaturizing the necessary technology while maintaining comfort and visual clarity in the lenses is an ongoing area of exploration [36, 40].

Beyond monitoring, wearable SCLs hold promise for therapeutic applications, particularly in the treatment of ocular diseases and conditions. Wearable SCLs can serve as advanced platforms for monitoring ocular biomarkers, enabling real-time assessment of eye health and early detection of diseases such as glaucoma and diabetic retinopathy. Additionally, these lenses can incorporate antimicrobial coatings to prevent infections, facilitate targeted drug delivery directly to the eye, and enable sensing and communication capabilities that allow users to receive health updates and alerts seamlessly [40]. For instance, these lenses are engineered so that could deliver medications directly to the eye over extended periods, improving compliance and therapeutic efficacy. The localized delivery of therapeutic agents can reduce systemic side effects, a common issue with traditional oral or injectable medications. Moreover, the potential for integrating phototherapeutic agents into these lenses opens new avenues for treating conditions such as glaucoma or age-related macular degeneration. Nonetheless, several challenges must be addressed to realize these therapeutic potentials. The formulation of biocompatible drug carriers that can be safely integrated into contact lenses is paramount, as is the ability to control the release rates of medications. Additionally, ensuring that the therapeutic agents remain stable and effective during prolonged wear requires careful consideration of the materials used in lens fabrication [36, 40].

Despite the promising applications, the successful adoption of wearable SCLs hinges on user acceptance and trust. Concerns regarding comfort, usability, and aesthetic appeal will influence whether individuals choose to integrate these devices into their daily lives. Moreover, incorporating advanced technologies, like artificial intelligence (AI) for data processing and decision-making, raises critical questions about data privacy and security. Users must be assured that their health information is protected from unauthorized access and misuse. To build user trust, manufacturers should prioritize transparency regarding data usage policies and incorporate robust encryption methods to safeguard sensitive information. Public education campaigns can also play a crucial role in informing potential users about the benefits and safety of these technologies, addressing misconceptions, and highlighting the importance of data privacy.

## MXene-Based Wearable (Bio) Electronics for Different Physiological Signals Monitoring

2D materials like graphene have emerged as promising candidates for wearable (bio) electronics due to their exceptional electrical conductivity, mechanical flexibility, and large surface area, enabling precise monitoring of various physiological signals, such as heart rate, electrocardiograms, and biochemical markers. Their lightweight and biocompatible nature allows for seamless integration into flexible sensors that can conform to the skin, facilitating continuous health monitoring and real-time data acquisition without compromising user comfort [41, 42]. In this context, MXenes and their composites have gained considerable attention in the field of wearable (bio) electronics due to their exceptional electrical conductivity, mechanical flexibility, and biocompatibility. These properties make MXene-based composites highly suitable for developing advanced wearable devices aimed at monitoring various physiological signals, such as heart rate, electrocardiogram (ECG), electromyogram (EMG), and even biochemical markers in sweat. MXene-based wearable devices can effectively monitor a range of physiological signals, providing real-time insights into an individual’s health status. For instance, when integrated into wearable sensors, MXenes can detect electrical signals from the heart, capturing ECG data with high fidelity. Their high conductivity allows for precise signal acquisition, which is essential for accurately assessing heart health and detecting arrhythmias or other cardiac conditions. Similarly, MXene sensors can be employed to monitor EMG signals from muscle activity, offering valuable information for rehabilitation and sports performance analysis. Moreover, MXene-based composites can also play a crucial role in biochemical sensing, such as analyzing sweat to monitor hydration levels, electrolyte balance, or metabolic states. The ability to detect specific biomarkers in sweat—such as glucose or lactate—could develop diabetes management or athletic performance optimization. However, the development of highly sensitive and selective biosensors requires ongoing research into functionalizing MXenes with specific receptors or enzymes to enhance their specificity for target analytes [43–46].

The advantages of MXene-based wearable electronics extend beyond their physical properties. The flexibility and lightweight nature of MXenes allow for comfortable wearability, which is essential for long-term monitoring and user acceptance. Unlike traditional rigid sensors, MXene devices can conform to the body’s contours, minimizing discomfort and improving data accuracy. Additionally, the ease of integration with other technologies, such as wireless communication systems, enables seamless data transmission to smartphones or cloud-based platforms, facilitating real-time health monitoring and analytics. Furthermore, MXenes exhibit excellent stability and durability, which are critical for wearable applications that require consistent performance over time. Their resistance to environmental factors, such as humidity and temperature, enhances their reliability, making them suitable for outdoor or athletic use [44, 47].

Despite their promising applications, several challenges must be addressed for the successful integration of MXene-based wearable electronics in physiological monitoring [44, 47]. One significant challenge is the scalability and reproducibility of MXene synthesis, which can hinder the mass production of devices. Moreover, researchers must ensure that MXene materials are thoroughly characterized and standardized to guarantee consistent performance across different batches. Another critical concern is the biocompatibility and long-term stability of MXenes when in contact with biological fluids. While MXenes have shown promise in laboratory settings, further studies are needed to assess their long-term effects on human tissues and their interaction with biological environments. Innovations in MXene functionalization could enhance sensor specificity, while advancements in wireless technology could improve data transmission and user interaction. Furthermore, integrating AI and machine learning algorithms can enhance data analysis, providing users with personalized insights and recommendations based on their physiological signals. This integration could lead to proactive health management, empowering individuals to take control of their health [48].

## MXene-Based Wearable SCLs for Healthcare Monitoring and Therapy

### Therapeutic Applications

Cataract surgery involves removing the affected lens of the eye and replacing it with an intraocular lens, thereby improving visual clarity. Unfortunately, this procedure results in the loss of accommodation, which is the lens’s ability to adjust focus dynamically. Various designs for accommodative intraocular lenses (AIOLs) have been proposed, but none have yet achieved effective clinical application [49]. Recent research has investigated two-dimensional Ti_3_C_2_T_x_ MXene as a transparent conductive electrode for AIOL applications. However, its potential role in mitigating excessive inflammation and facilitating wound healing post-surgery remains underexplored. Following cataract surgery, chronic inflammation can arise, promoting epithelial-mesenchymal transition (EMT) in the remaining lens epithelial cells that can led to the development of a fibrotic tissue on the posterior capsule, commonly referred as posterior capsule opacification (PCO). Because of its high surface area and surface functionalization capability, Ti_3_C_2_T_x_ MXene shows potential for dual-function AIOL designs, potentially helping to inhibit pathways involved in the development of PCO. The effects of Ti_3_C_2_T_x_ on chronic inflammation and EMT pathways were examined in an in vitro model of lens epithelial cells [49]. In this study, different assays were performed for comprehensive gene expression profiling, and lipidomics analysis, including scratch assays, enzyme-linked immunosorbent assays (ELISA), RNA sequencing, and quantitative polymerase chain reaction (qPCR). The results indicated that presence of MXene could significantly decreased pro-inflammatory cytokines levels in interleukin 1 beta (IL-1*β*)-primed LECs without affecting EMT, thereby promoting a conducive environment for wound repair [49]. This study highlights the potential of MXenes within AIOL designs, suggesting their ability to inhibit critical pathways associated with PCO development.

**Fig. 2:**
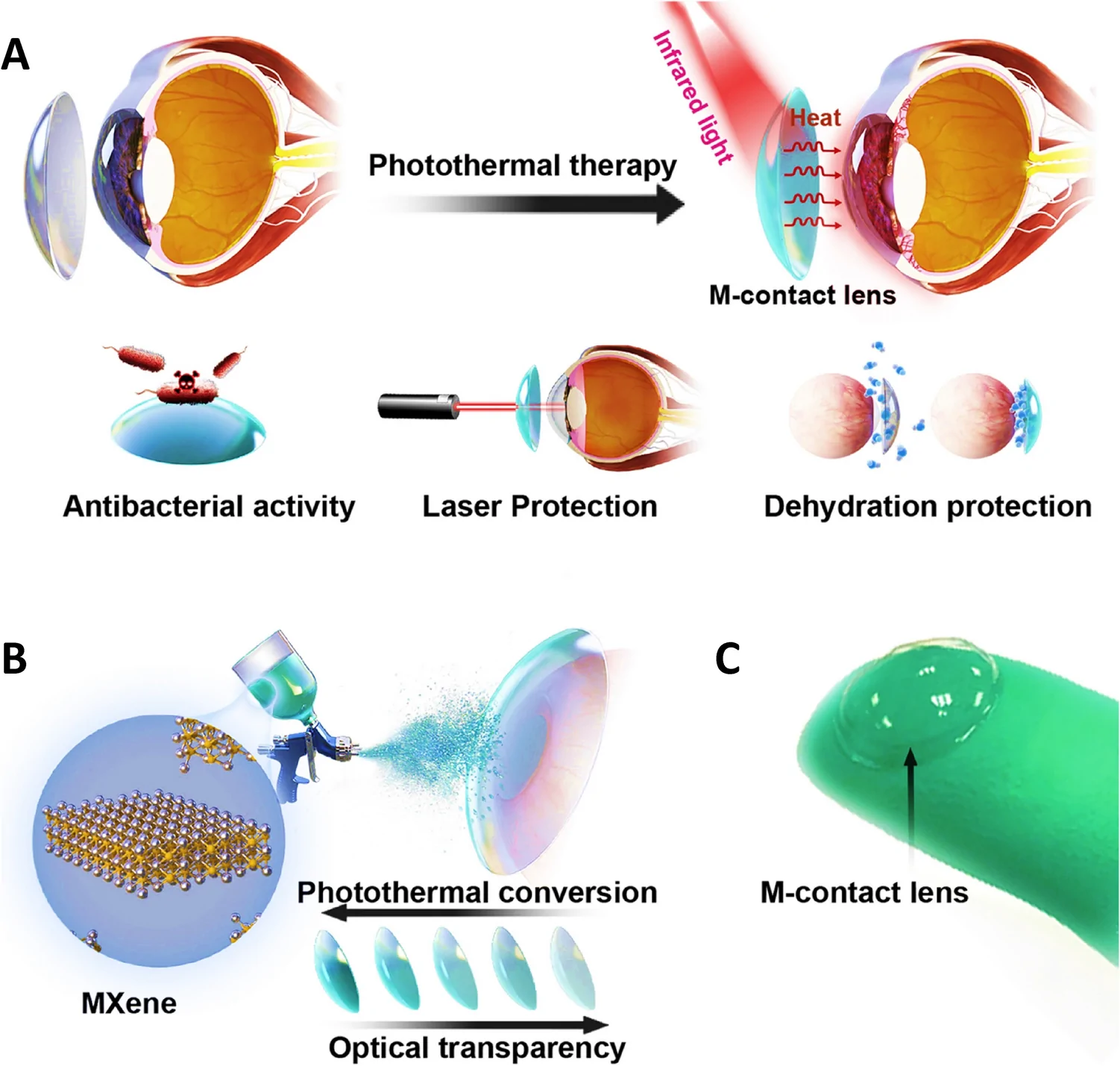
**A** Use of MXene-coated contact lenses, referred to as M-contact lenses, in ocular PTT is an innovative approach. This process involves the transfer of heat from the contact lens to the front part of the eye, highlighting the transformation of blood vessels before and after the treatment. Additionally, **B** details regarding the preparation of the M-contact lens are provided, **C** along with its digital representation. Reproduced with permission from Ref. [50] Copyright 2023 Elsevier

Jin et al. [50] achieved notable progress in ocular technology by creating a transparent, multifunctional contact lens designed for wearable use. This innovative design utilized Ti_3_C_2_T_x_ MXene, which was spray-decorated onto a standard commercial contact lens (Fig. 2). This advancement not only enhanced the functionality of the lens but also opened new avenues for ocular photothermal therapy (PTT) and eye protection. Consequently, the exceptional photothermal conversion efficiency was achieved in both in vitro and in vivo tests, particularly in rabbit eyes. In here, utilizing the infrared irradiation in combination with MXene-coated contact lens led to a significant enhancement in vascular blood flow of the ocular anterior segment, suggesting a potential therapeutic effect. This is particularly important as improved blood flow can facilitate healing and recovery in various eye conditions. Moreover, the multifunctional properties of the lens extend beyond just photothermal therapy. It exhibited excellent laser protection capabilities, safeguarding the eyes from harmful radiation, as well as moisturizing effects that can enhance user comfort. The antibacterial properties of the MXene-adorned contact lens were equally impressive. In the experiments, no *Staphylococcus aureus* colony was observed on lens-derived cultures, indicating its effective antibacterial action. Additionally, this lens demonstrated significant antibacterial effects against *Escherichia coli* [50]. Thus, the incorporation of MXenes into contact lenses represents a major leap forward in ocular health technology. These multifunctional lenses not only enable targeted photothermal therapy but also provide robust protection against harmful UV radiation and bacteria. This dual functionality underscores the potential of MXenes in enhancing wearable technology, paving the way for future advancements in ocular care and beyond. Experimental data confirm that MXene-based wearable contact lenses substantially enhance the therapeutic, protective, and comfort properties of traditional contact lenses. They offer superior blood flow improvement, antibacterial and anti-inflammatory effects, and maintain high optical clarity and biocompatibility, making them highly promising for clinical applications in eye disease therapy and vision care.

## Improving Vision

**Fig. 3:**
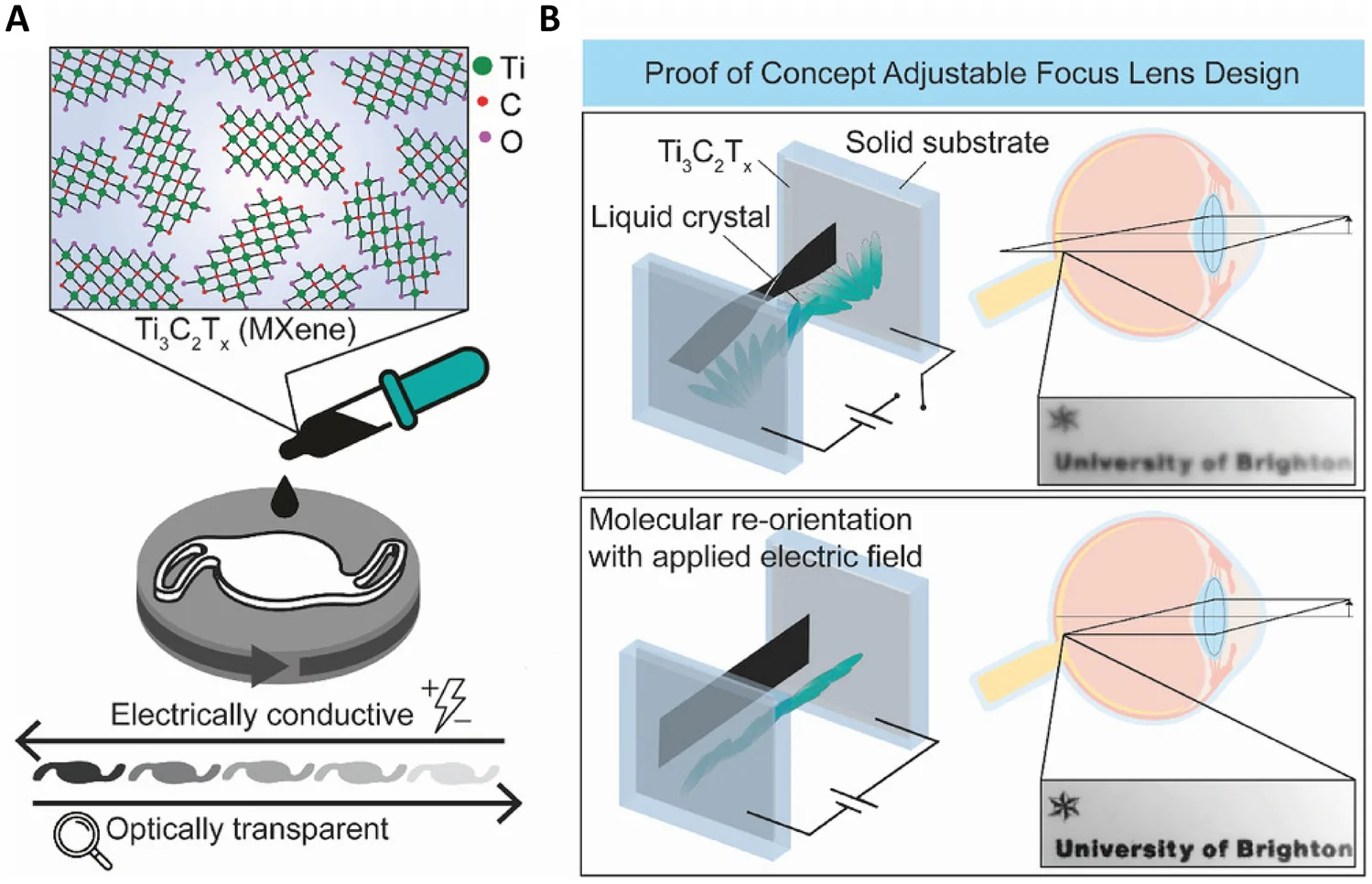
**A** This section illustrates the design of an AIOL featuring MXene applied as a spin-coated layer on an acrylate IOL from Rayner Intraocular Lenses Ltd, UK. The modified lens exhibits essential characteristics such as excellent electrical conductivity and high optical transparency. **B** A prototype adjustable-focus lens was developed as a proof of concept. The constructed test cell consisted of glass slides coated with MXene, which sandwiched a liquid crystal layer (5CB) oriented in a twisted nematic configuration. In the absence of an electric field, the image remained blurred. However, applying an electric field triggered molecular reorientation within the liquid crystal layer, bringing the image into clear focus. Reproduced from Ref. [51] under the terms of the Creative Commons CC BY license

The application of MXenes in ocular devices could lead to significant improvements in visual restoration technologies. The Ti_3_C_2_T_x_ MXene, as a transparent and conductive electrode, was synthesized to enable the modulation of optical power (Fig. 3) [51]. The synthesis of Ti_3_C_2_T_x_ involved a spin-coating process applied to hydrophobic acrylate IOLs. The fabricated electrodes exhibited sheet resistance values between 0.2 and 1.0 kΩ sq^−1^, along with visible light transmittance ranging from 50 to 80%. Importantly, tests on human lens epithelial and monocytic cells revealed no signs of cytotoxicity or inflammation associated with the coated lenses. To further explore the capabilities of Ti_3_C_2_T_x_, an adjustable focus test cell was created. This cell incorporated a liquid crystal layer positioned between two Ti_3_C_2_T_x_ coatings on a stable support. Utilizing an electric field induced a shift in the molecular orientation of the LC layer, leading to alterations in optical power. As a result, objects observed through the test cell alternately appeared sharp and blurred, indicating the feasibility of tunable vision correction. This study exhibited a foundational step toward integrating MXene into the design of accommodating IOLs, showcasing the ability for reversible and controlled adjustments in focus [51].

## Sensing and Monitoring

**Fig. 4:**
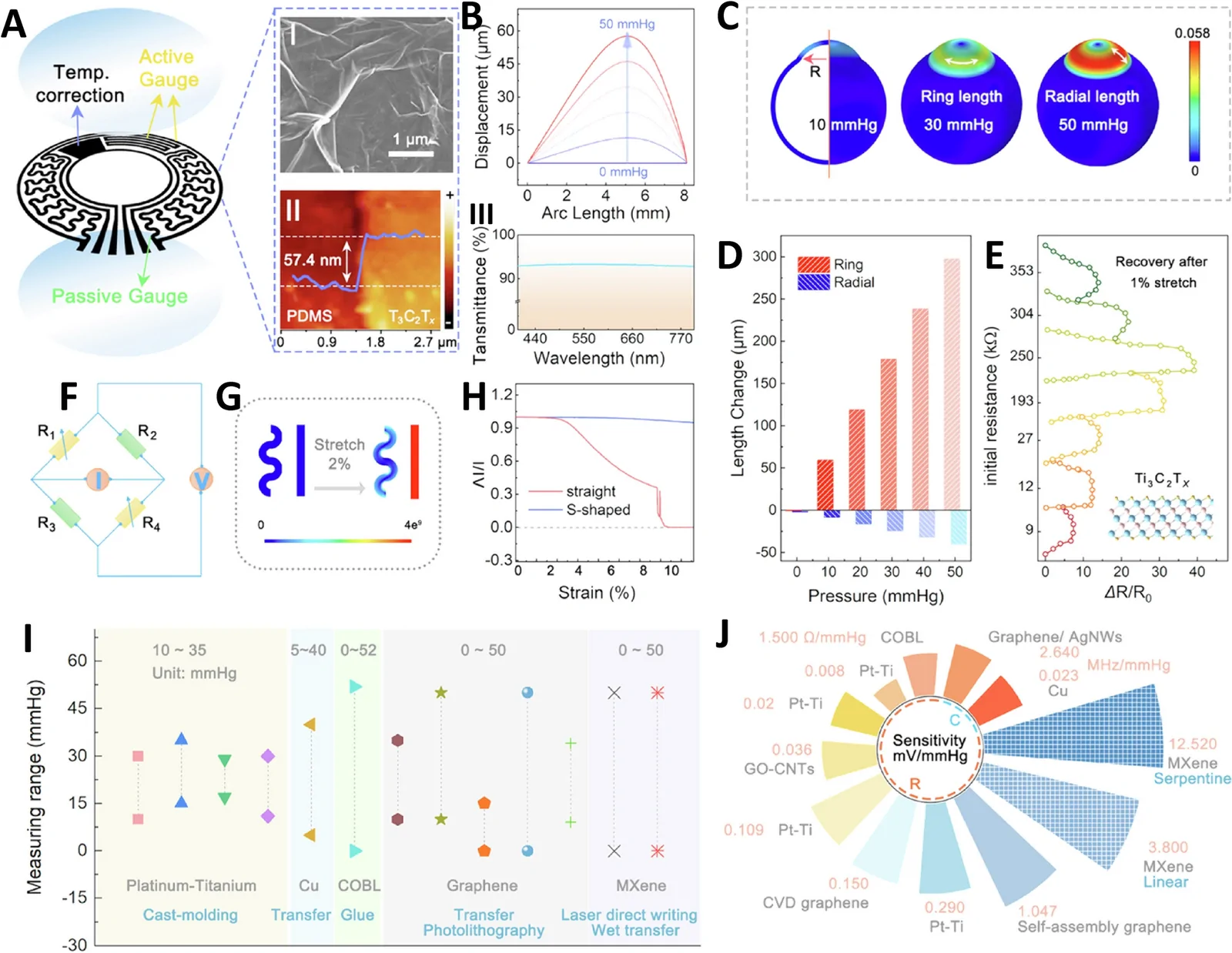
**A** Arrangement of the Ti_3_C_2_T_x_-SCL is depicted in various panels. (I) SEM image and (II) AFM results of Ti_3_C_2_T_x_ MXene electrode. (III) The Ti_3_C_2_T_x_-coated soft contact lens exhibited significant transparency in the viewing region. **B** There is a correlation between the corneal basal arc at varying distances from axial deformation (R) and intraocular pressure (IOP). **C** Surface stress distribution on the eyeball changes with varying intraocular pressures (10, 30, and 50 mmHg), showing the cornea in the upper part and the sclera in the lower part. The cornea’s circumferential and radial directions are clearly indicated. **D** Changes in IOP are associated with deformation of the corneal base arc along both the ring and radial directions. **E** Evaluation of electrode thickness effects on sensitivity during 1% strain recovery (in the Ti_3_C_2_T_x_ MXene structure blue, pink, and yellow spheres are demonstrating the titanium atoms, carbon atoms, and surface functional groups, respectively). The schematic images of **F** Wheatstone bridge circuit (in which R1 and R4 were acted as active gauges, while R2 and R3 were used as passive gauges) and **G** Finite element analysis evaluated the behavior of rectilinear and serpentine electrodes in both their initial state and when subjected to 2% strain. **H** Results of changes occurred in current in response to the tensile strain for both straight and S-shaped electrodes. Comparing the results of **I** measurement range and **J** sensitivity of the fabricated Ti_3_C_2_T_x_ Wheatstone bridge-structured IOP strain sensor with previous sensor. Pt-Ti: platinum-titanium, GO-CNTs: graphene oxide, and carbon nanotubes, COBL: conductive all-organic bilayer film. Reproduced from Ref. [53] under a Creative Commons Attribution 4.0 International License (CC BY)

There has been a growing interest in wearable soft contact lens sensors for real-time, noninvasive monitoring of intraocular pressure (IOP), especially in patients with glaucoma or those recovering from myopia surgery [52]. As the prevalence of eyestrain increases, the need for innovative solutions becomes even more pressing. In response to this challenge, an innovative smart closed-loop system was developed, combining a Ti_3_C_2_T_x_ MXene-based soft contact lens sensor with wireless communication modules, a visual display, and alert functionalities. This cohesive system enabled users to monitor IOP continuously and in real time without causing discomfort or harm to the eyes. The MXene-based soft contact lens sensor demonstrated outstanding performance, with a high sensitivity of 7.483 mV mmHg^-1^, strong linearity on silicone eyeball models, and notable stability throughout extended pressure-release testing. Furthermore, the lens maintained a transparency of 67.8% under visible light, ensuring that users experience minimal obstruction to their vision. Biocompatibility studies revealed that the MXene-based soft contact lens sensor could be comfortably worn on rabbit eyes with no discomfort reported. When paired with a wireless module, the system allowed users to monitor IOP and received alerts through their smartphones. This innovative approach not only highlighted the potential of Ti_3_C_2_T_x_ materials in creating multifunctional contact lens-based sensors but also paved the way for advanced continuous and nondestructive IOP measurement systems, enhancing patient care and outcomes in ophthalmology [52]. In another study, an innovative neuroprosthetic contact lens was developed. This remarkable device incorporated a sophisticated sensorimotor system, featuring a Ti_3_C_2_T_x_ Wheatstone bridge-structured IOP strain sensor alongside a Ti_3_C_2_T_x_ temperature sensor (Fig. 4) [53]. Together, these components worked seamlessly with an IOP point-of-care monitoring and display system. One standout feature of this neuroprosthetic contact lens was its impressive sensitivity, recorded at 12.52 mV mmHg^−1^. This high sensitivity enabled real-time monitoring and timely alerted regarding IOP fluctuations. Remarkably, in vivo studies conducted on rabbit eyes revealed the lens’s excellent wearability and biocompatibility. Moreover, further experiments involving live rats successfully simulated the biological sensorimotor loop. Notably, researchers observed leg twitching at various angles, controlled by the motor cortex in response to somatosensory cortex commands. This twitching occurred when IOP deviated from the normal range, highlighting the lens’s potential to facilitate responsive treatment [53].

**Fig. 5:**
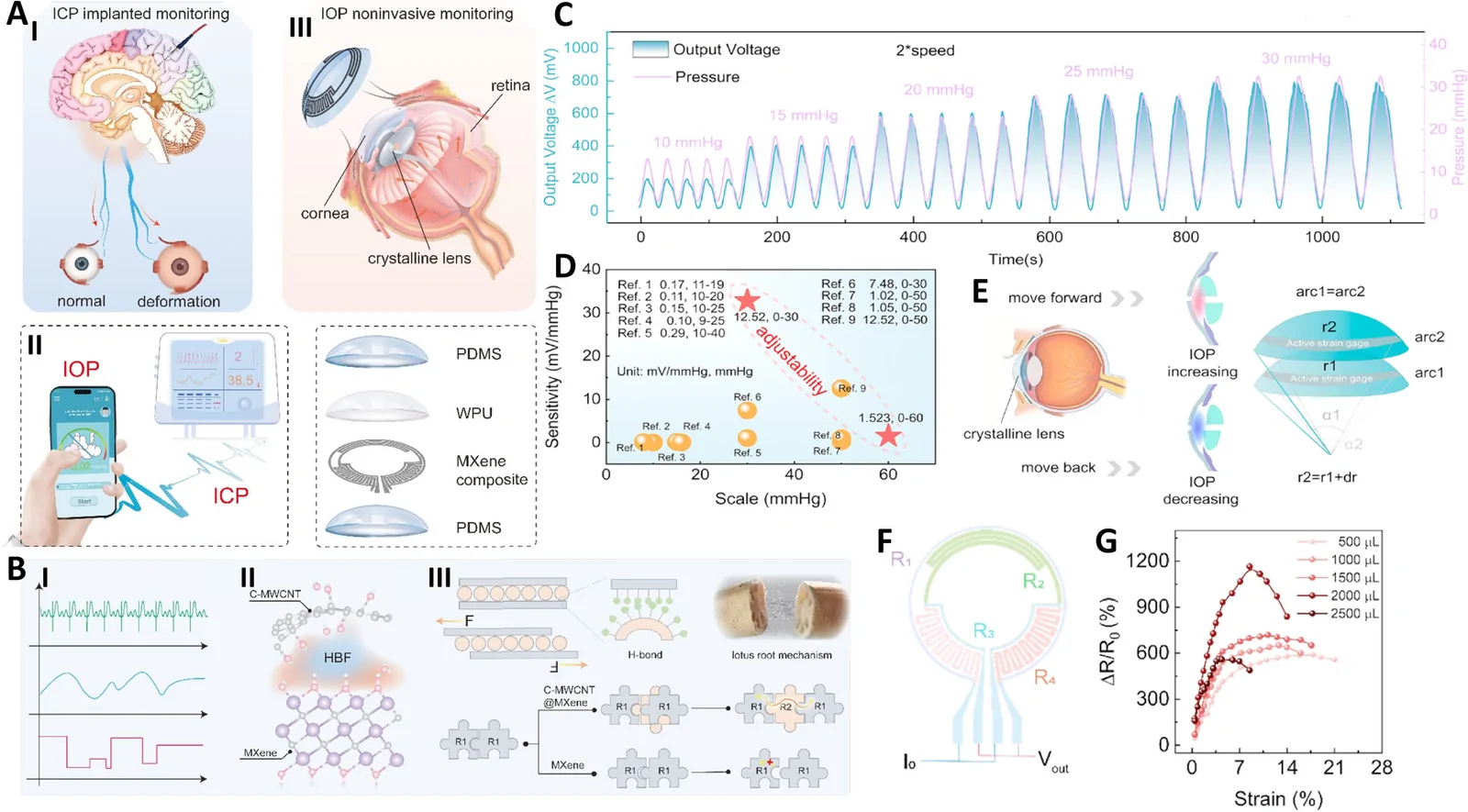
**A** Schematic image related to the current method used for detection of ICP (I), correlation between ICP and IOP detection (II), and application of wearable smart MXene based contact lens used for the detection of IOP (III). **B** Schematic images of (I) the alignment and propagation of IOP signals (red), ICP signals (blue), and circadian rhythm signals (green), (II) the formation of hydrogen bonds between carboxylic acid groups of C-MWCNTs and surface functional groups of MXene, and (III) mechanism of lotus root mimic IOP sensor, in which R₁ reflectd the combined electrical impedance from both Ti₃C₂Tₓ MXene and C-MWCNTs, analogous to the two ends of a lotus root, while R₂ corresponded to the elongated C-MWCNT strands produced when microcracks form under applied pressure. **C** Results of using different pressures on output voltage of fabricated IOP sensor. **D** Comparing the sensitivity of fabricated sensor with other research studies. **E** Schematic image of changes occurred in corneal shape and corresponding deformation of the contact lens in response to alteration in IOP. **F** Schematic image of the Wheatstone bridge circuit in the fabricated sensor. **G** The sensitivity results of fabricated sensor in response to different electrode thicknesses. Reprinted with permission from [57]. Copyright 2025, American Chemical Society

Intracranial pressure (ICP) is an important factor affecting patients undergoing surgery or trauma, as well as athletes, astronauts, and even healthy individuals in weightless environments. Accurate ICP monitoring can facilitate treatment decisions, optimize training for high-performance individuals, and support evidence-based healthcare. However, conventional ICP monitoring typically requires invasive devices that risk causing brain damage, which is particularly unsuitable for athletes and astronauts. Interestingly, studies have found a link between ICP and intraocular pressure (IOP), as changes in ICP can influence the pressure in the eye’s anterior chamber. This opens the possibility of using noninvasive, eye-wearable IOP sensors for continuous ICP monitoring [54–56]. This was test in a recent study that produced a type of eye-wearable sensor that mimic the lotus root structure to measure changes in the IOP through the microcrack strain sensing mechanism. The fabricated sensor was a sandwich structure composed of four main layers including polydimethylsiloxane (PDMS) layer, a composite of Ti_3_C_2_T_x_ MXene@carboxylated carbon nanotube (C-MWCNT), and a waterborne polyurethane (WPU)/PDMS layer (Fig. 5). The strong hydrogen bonding interaction, occurred between the abundant –COOH groups on C-MWCNTs and the polar functional groups of MXene, led to enhance the performance of stress sensors. Based on this hydrogen bonding, a novel microcrack network resembling a lotus root structure was designed, enabling the development of a stable and highly sensitive IOP sensor with a broad sensing range and tunable sensitivity. Moreover the hydrophilicity feature of the WPU facilitated the uniform deposition of aqueous Ti₃C₂Tₓ MXene onto its surface via spraying. When coated on PDMS, WPU improved the mechanical properties of the film, for instance it led to lowering the Young’s modulus, increasing fracture elongation, and providing excellent elasticity and shape recovery under tensile strain. The incorporation of C-MWCNTs induced surface folding and improved structural integrity. The fabricated sensor exhibited broad detection range (up to 60 mmHg) and high sensitivity (33.21 mV mmHg^−1^) that enabled accurate, real-time monitoring of IOP. Tests conducted on live rabbits, either wearing the sensor or implanted with a commercial ICP probe, revealed strong alignment in recorded data across various body positions that confirmed the practicality of using the eye-wearable IOP sensor for noninvasive ICP monitoring, with promising applications in cerebrovascular and ophthalmic diagnostics, clinical care, and astronaut training [57].

Ocular devices designed to be worn on the human eye, featuring a hemispherical shape and intelligent functionalities, hold immense potential for continuous and nondestructive management of oculopathy diseases [58]. Given the wealth of physiological and biochemical information present in human eyes, these devices can play a critical role in monitoring eye health. However, the fabrication and integration of various devices onto a hemispherical surface with its unique curvature present significant engineering challenges [58]. In this context, the combination uses of strain sensors and a micro-supercapacitor led to the production of a Ti_3_C_2_T_x_ MXene-based hemispherical integrated system that was specifically applied for IOP monitoring. The innovative approach of utilizing Ti_3_C_2_T_x_ as both electrode material for the micro-supercapacitor and piezoresistive films for the strain sensors simplified the manufacturing process significantly. This integration not only reduced complexity but also enhanced the overall performance of the device. The MXene-based micro-supercapacitor exhibited impressive characteristics, with a high specific capacitance of 32 mF cm^−2^ at a 5 mV s^−1^ scan rate and 10 mWh cm^−2^ energy density, ensuring a stable output voltage capable of driving the sensor. In addition, the fabricated sensor displayed exceptional sensitivity of about 0.014 mmHg^−1^ within the range of 0–50 mmHg, facilitating continuous IOP monitoring. The self-powered hemispherical integrated device offered quick and consistent responses to varying IOP levels, demonstrating great potential for diagnosing and treating eye diseases, ultimately advancing the field of ophthalmic health management [58].

## Enhancement of Performance

Contact lenses often face significant challenges due to the adhesion and invasion of pollutants and pathogenic bacteria, which can lead to infections and inflammatory diseases [59]. To address these issues, the functionalization of contact lenses with biological functions—such as anti-fouling, antibacterial, and anti-inflammatory properties—while preserving their transparency remains a critical goal. In this innovative study, vanadium carbide (V_2_C), a member of the MXenes family, was utilized to enhance the performance of contact lenses [59]. Through a water transfer printing method, a uniform film of V_2_C was created on the contact lens surface, leveraging the Marangoni effect to ensure a tightly arranged coating. This approach resulted in a stable interface due to the electrostatic forces, effectively integrating the V_2_C into the contact lens material. The V_2_C-modified contact lens exhibited a remarkable combination of optical clarity and functional properties. It demonstrated strong biocompatibility, impressive antioxidant capabilities, and significant anti-inflammatory activities. *In-vitro* antibacterial tests showcased the excellent performance of V_2_C-modified contact lens in preventing bacterial adhesion, effectively sterilizing surfaces, and inhibiting biofilm formation. These attributes were particularly advantageous in the treatment of infectious keratitis, as V_2_C-modified contact lens not only eliminated bacteria but also alleviated inflammation. Overall, this advancement in contact lens technology highlighted the potential of MXenes like V_2_C to enhance the safety and effectiveness of wearable optical devices, paving the way for healthier and more reliable options for contact lens users [59].

Due to their high excellent electrical conductivity, MXenes could act as an ideal electromagnetic (EM) shielding compound in the structure of contact lenses that are used for the protection of eyes from radiation-induced eye diseases. This was approved in a recent research, in which multilayered Ti_3_C_2_T_*x*_ MXene (in combination with cellulose ester) were applied as a coating film on the surface of a type of commercial contact lens using acetone treatment to provide it for physical attachment. Utilizing acetone wash led to provide antioxidant property for MXene layer, as well, so that could protect this layer from oxidation when exposed with the atmosphere. Assessing the effect of MXene concentrations on the transparency and conductivity of final product showed a direct relationship with the concentration, so that by increasing the concentration the conductivity and thickness of the MXene layer were increased as well, and at the same time, the transparency decreased. It also showed enhancement in dehydration protection of lens so that could reduce (~ 36%) the water vapor transmission rate (WVTR) of the commercial lens. The effect of MXene layer on the electromagnetic interference (EMI) shielding effect was also checked ex vivo using 170 W microwave oven for 30 s. Results showed increasing in the temperature of porcine eyes in both cases; however, the eyes treated with the commercial lens showed higher increment (45 °C compared with 36 °C in commercial and MXene coated lenses, respectively). Moreover, the temperature of the lenses was also checked after exposing them with EM radiation (170 W, for 30 s) that showed increasing in the temperature of MXene coated lens, while the commercial type had no increment, all of which confirmed the EMI shielding effect of MXene. Results of in vitro and in vivo biocompatibility assessments also confirmed the safety of fabricated lens without showing any significant inflammatory reaction that affirm the potential of fabricated lens for further wearable applications [60].

## Challenges

The development of wearable contact lens biosensors also comes with its fair share of challenges. One significant hurdle is ensuring the accuracy and reliability of the collected data. Factors such as tear composition, environmental conditions, and user-specific variables can influence sensor performance and lead to potential inaccuracies [61]. Overcoming these challenges requires rigorous calibration and validation processes. Another challenge lies in the integration of power sources and wireless communication capabilities into the compact form factor of contact lenses. Energy-efficient solutions, such as miniaturized batteries or energy harvesting technologies, need to be explored to power these biosensors without compromising comfort or hindering vision. One limitation of wearable contact lens biosensors is the need for frequent replacement to maintain accuracy and functionality. Contact lenses have a limited lifespan, and the sensors embedded within them may degrade or become less effective over time. This necessitates regular replacement, which can add to the overall cost and maintenance of these devices [1, 2].

The eye serves as a rich source of biomarkers for various diseases and health conditions, encompassing both chemical and physical factors. Notably, substances like glucose, uric acid, and cholesterol are found in high concentrations in tears, prompting the development of numerous sensors for their measurement [62]. However, many potential biomarkers exist in trace amounts, necessitating more accurate and sensitive sensing technologies for detection. To overcome these limitations, researchers are investigating advanced sensing materials that offer enhanced sensitivity and specificity. For instance, innovative materials such as enzymes, aptamers, and antibodies have been employed to bolster the performance of electrochemical sensors aimed at detecting target biomarkers. Moreover, the integration of machine learning and AI technologies has further elevated the capabilities of SCLs as a diagnostic platform [63]. By employing pattern analysis of biomarker fluctuations, these technologies can significantly improve the accuracy and sensitivity of disease detection. The combination of advanced sensing materials and intelligent analytical methods holds great promise for expanding the utility of ocular biomarkers, paving the way for more effective disease diagnosis and therapeutic interventions through wearable technology [1, 62].

Breaking through clinical barriers is crucial to addressing the current challenges associated with contact lens technology. Key issues, such as ensuring comfort, durability, and safety for long-term wear, remain significant hurdles. Overcoming these manufacturing challenges is essential for the widespread acceptance and accessibility of these advanced technologies in clinical settings. Furthermore, the accuracy and reliability of measurements are vital for effective disease diagnosis. To tackle these concerns, robust sensor technology, coupled with advanced algorithms for data analysis, must be developed and validated through rigorous clinical trials. Creating an environment with low barriers to clinical trials is essential for the effective development of SCLs. This involves streamlining regulatory processes and fostering collaboration between researchers, manufacturers, and regulatory bodies. Additionally, as SCLs move toward commercialization, establishing systems related to personal information protection and adhering to medical device regulations will be paramount. By prioritizing these aspects, the pathway to bringing innovative SCL technology to market can be optimized, ultimately improving patient outcomes and enhancing the efficacy of ocular health monitoring and disease diagnosis [1, 64].

**Fig. 6:**
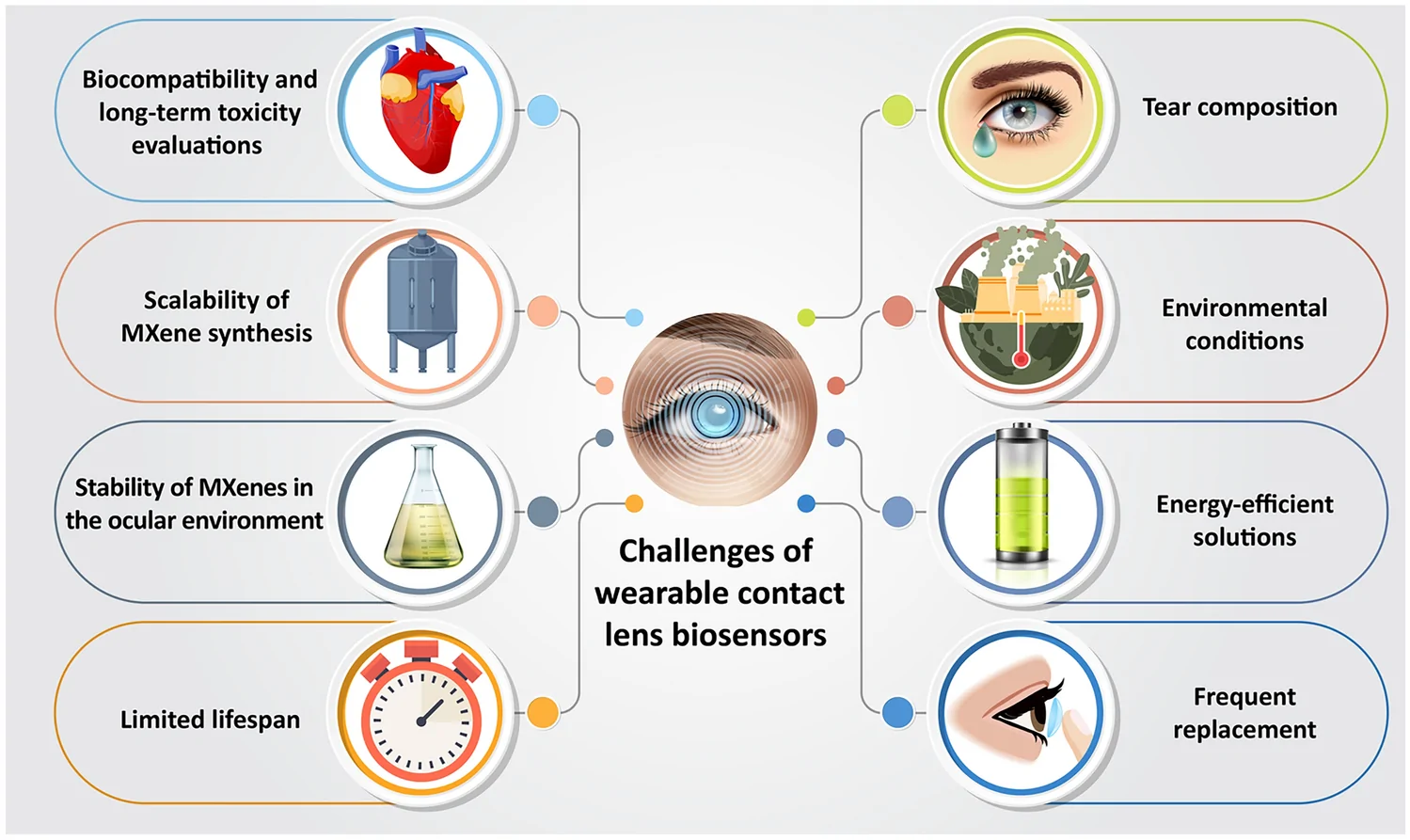
Some of the important challenges of MXene-based wearable SCLs

The development of MXene-based contact lenses faces several intrinsic and practical challenges that must be addressed to meet the specific requirements of ocular devices (Fig. 6). One significant challenge is the stability and oxidation of MXenes, such as V₂C or Ti₃C₂, which are prone to degradation when exposed to oxygen and moisture. This instability poses a risk for contact lenses, as they need to maintain consistent performance and safety during prolonged wear. Finding suitable surface coatings or protective layers to shield MXenes from the corrosive effects of tear fluid is an important consideration. Another challenge is the scalability of MXene synthesis. Additionally, ensuring the long-term stability of MXene coatings under physiological conditions, such as interactions with tear fluid and mechanical forces from blinking, remains a critical hurdle. Another pressing issue is maintaining optical transparency; MXene coatings must be thin and uniform to achieve over 80% light transmittance without causing haze or reduced clarity, which can impair vision and overall wearer comfort. Additionally, MXenes demonstrate promising antibacterial and anti-adhesion properties that could help combat bacterial infections like keratitis, but the effectiveness of these properties needs validation in real-world conditions. The coatings must also be mechanically durable, exhibiting strong adhesion to lens substrates like silicone hydrogel while resisting stress from blinking and handling.

Biocompatibility and long-term toxicity evaluations are important aspect when using MXenes in contact lenses. Thorough studies are necessary to evaluate any potential adverse effects or interactions between MXenes and ocular tissues. Ensuring that MXene-based contact lenses do not cause irritation, inflammation, or other ocular complications is crucial for their safe and effective use. It’s essential that these coatings do not disrupt natural tear film dynamics or protein interactions necessary for cell health. MXenes and their composites show suitable biocompatibility, although their cytotoxicity varies depending on factors such as size, dose, surface chemistry, and cell type. Smaller MXene nanosheets and quantum dots tend to exhibit different toxicological profiles compared to larger sheets. For instance, Ti₃C₂Tₓ MXene nanosheets demonstrated low toxicity at concentrations below ~ 50 μg/mL, but higher doses can considerably reduce cell viability, especially in sensitive cells like human mesenchymal stem cells (hMSCs) [65, 66]. Surface modifications, such as polymer coatings, can enhance their biocompatibility by improving stability and minimizing harmful interactions with cell membranes. Moreover, MXenes are generally more toxic to cancer cells than to normal cells, which is advantageous for anticancer applications. In vitro studies typically show that MXenes maintain cell viability above 70%–80% at low concentrations, but higher doses can lead to reduced viability and altered metabolic functions. Limited in vivo studies suggest acceptable biocompatibility at controlled doses, though concerns regarding long-term accumulation and potential immunomodulatory effects remain. To mitigate toxicity, strategies like surface functionalization and optimizing dosages are essential, while ensuring stability in physiological environments is crucial to prevent the formation of toxic byproducts. Ongoing research, including machine learning approaches to predict cytotoxicity, aims to enhance the safe design and application of MXenes in biomedical fields [66, 67].

Currently, MXenes are primarily produced in small quantities in laboratory settings. Scaling up the production process while maintaining the desired properties and quality is crucial for practical implementation in wearable contact lenses. In this context, the scalability and reproducibility of manufacturing MXene-coated contact lenses pose challenges, as current synthesis methods often involve hazardous chemicals and must be integrated into existing production lines without compromising lens quality or increasing costs. Fluorine, often introduced during the synthesis of MXenes through fluoride-containing etchants like HF or LiF/HCl, significantly influences the surface chemistry and biocompatibility of these materials [68]. The presence of residual fluoride ions raises concerns for biomedical applications due to their potential cytotoxic effects and ability to provoke inflammatory responses in biological systems. Even low concentrations of fluoride can disrupt cellular processes, leading to toxicity, especially when MXenes are used in direct contact with cells or tissues. Additionally, the fluorinated surfaces can hinder protein adsorption, which is critical for cell attachment and integration with biological tissues, ultimately diminishing the biocompatibility of MXenes compared to those fully functionalized with more favorable groups like hydroxyl. To overcome these challenges, recent research has focused on developing fluorine-free synthesis methods for MXenes, such as electrochemical etching and hydrothermal processing with alkali solutions. These techniques can produce MXenes without introducing fluorine, thereby enhancing their safety profile for biomedical applications. Post-synthesis surface modifications can further mitigate cytotoxicity by replacing or masking residual fluorine with more biocompatible functional groups. While the use of fluorine-containing etchants poses significant challenges regarding the biocompatibility of MXenes, advances in synthesis techniques and surface modifications are actively addressing these issues, making MXenes more suitable for a range of biomedical applications [65, 68–70].

## Future Perspectives

**Fig. 7:**
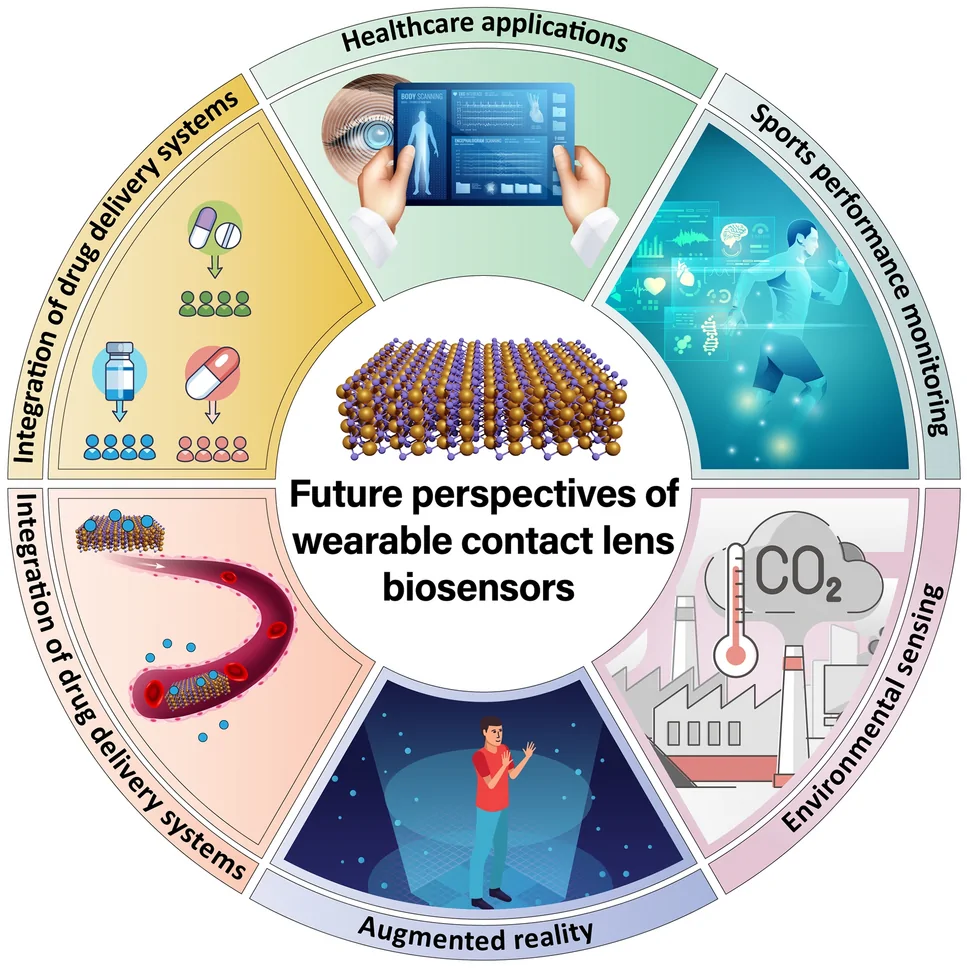
Future perspectives of MXene-based wearable smart contact lenses

Despite the challenges and limitations, wearable contact lens biosensors offer numerous advantages [71]. Their non-invasive nature eliminates the discomfort and inconvenience associated with traditional monitoring methods. They provide real-time data, allowing for immediate intervention or adjustments based on the collected information. Moreover, their portability and unobtrusiveness make them highly convenient for users, enabling continuous monitoring without disrupting daily activities. Additionally, the integration of AI algorithms and machine learning techniques can enhance the interpretation and analysis of the collected data, leading to more personalized and precise healthcare interventions [3]. The potential expansion of biosensors to detect a wider range of biomarkers and physiological parameters opens up opportunities for early diagnosis and prevention of various diseases. Furthermore, wearable contact lens biosensors may find applications beyond healthcare. They could be utilized in sports performance monitoring, environmental sensing, and augmented reality, among other fields. Some of the important future prospects have been added in Fig. 7.

One of the key advantages of wearable contact lens biosensors is their ability to provide continuous monitoring [72]. Unlike traditional monitoring methods that require intermittent measurements, these biosensors offer a constant stream of data, allowing for a more comprehensive understanding of an individual’s health status. This continuous monitoring can enable early detection of changes or abnormalities, leading to timely intervention and improved health outcomes. Moreover, wearable contact lens biosensors have the potential to develop personalized medicine. By collecting real-time data on various physiological parameters, these devices can provide valuable insights into an individual’s health trends and patterns. This information can be used to tailor treatment plans, medication dosages, and lifestyle recommendations specifically to each person’s needs [72].

With advances in nanotechnology, researchers are exploring the incorporation of additional functionalities into these lenses [71]. For instance, the integration of drug delivery systems within the lens structure could enable targeted and controlled release of medications directly onto the eye, further enhancing the management of ocular and systemic diseases [12]. Another area of future development lies in the field of augmented reality. Wearable contact lens biosensors could serve as a platform for augmented reality applications, overlaying digital information onto the user’s field of vision. This opens up possibilities for enhanced visual experiences, improved navigation, and augmented healthcare interventions, such as providing real-time health data or guidance during medical procedures.

The incorporation of visible readouts into MXene-based smart contact lenses could develop the way users interact with their health data. These readouts can provide real-time visual feedback on various physiological parameters, such as intraocular pressure, glucose levels, or other biomarkers, directly through the lens. This feature enhances user engagement and promotes proactive health management, as individuals can monitor their conditions without the need for external devices. For instance, visible indicators could alert users to potential health issues, facilitating timely intervention. However, the challenge lies in developing transparent, lightweight displays that do not compromise comfort or vision. Research must focus on optimizing the optical properties of MXenes while ensuring that the embedded technology remains user-friendly and unobtrusive.

AI has the potential to significantly enhance the performance of MXene-based smart contact lenses. By integrating AI algorithms, these lenses can analyze data collected from users in real-time, providing personalized insights and recommendations based on individual health metrics. For example, AI could predict fluctuations in glucose levels in diabetic patients or identify patterns linked to ocular diseases, thereby enabling personalized treatment plans. Additionally, machine learning could improve the accuracy of sensors within the lenses, enhancing their ability to detect critical changes in health parameters. However, the effectiveness of AI integration hinges on the quality of data collected and the robustness of the algorithms employed. Ensuring the accuracy and reliability of AI-driven insights will require extensive validation and testing, alongside ongoing collaboration between engineers, healthcare professionals, and data scientists.

As MXene-based smart contact lenses become increasingly interconnected and data-driven, concerns regarding data privacy and security must be addressed. The collection and transmission of sensitive health information pose significant risks, particularly if proper safeguards are not implemented. Users must be assured that their personal data is protected from unauthorized access and breaches. To mitigate these risks, developers should prioritize the incorporation of advanced encryption methods and secure data storage solutions. Furthermore, transparency in data usage policies is essential; users should be informed about how their data will be utilized and who will have access to it. Establishing a robust framework for data privacy will not only foster user trust but also comply with regulatory standards, paving the way for broader acceptance of these innovative technologies in the healthcare market.

## Conclusion

MXenes have recently emerged as transformative components in the realm of smart wearable technologies, particularly in contact lenses. These materials, characterized by their unique electrical, thermal, and mechanical properties, present a myriad of advantages for enhancing the functionality of SCLs. By incorporating MXenes, designers can create lenses that not only monitor health metrics but also respond dynamically to environmental stimuli. For instance, the electrical conductivity of MXenes enables the integration of sensors directly into the lens, allowing real-time tracking of ocular parameters such as intraocular pressure and glucose levels. Moreover, MXenes can be combined with various polymers, resulting in composites that improve the structural integrity and flexibility of the lenses. This combination allows for the creation of lightweight and durable smart lenses, which can maintain user comfort throughout the day. Notably, these composites can exhibit enhanced transparency, preserving the aesthetic appeal of traditional contact lenses while embedding sophisticated technology.

While MXenes present thrilling opportunities for the evolution of wearable contact lenses, several significant challenges must be addressed to realize their full potential. Stability is a primary concern; the performance of MXenes can degrade under various environmental conditions. This instability may affect the longevity and reliability of smart lenses. Furthermore, biocompatibility poses another hurdle. Ensuring that MXenes do not provoke adverse reactions within the eye is essential for user safety. Scalability also remains a critical issue. The production of MXene-based composites requires advanced manufacturing techniques, which can be costly and complex. As demand for smart lenses grows, developing efficient, cost-effective production methods will be vital. Despite these obstacles, the future of MXene-based wearable contact lenses is promising. With additional explorations, these lenses could develop vision correction and ocular healthcare. They may offer enhanced functionalities, such as real-time health monitoring and personalized treatment options. The convergence of advanced materials and innovative design has the potential to reshape how we approach vision and overall ocular health, ushering in a new era of smart wearables.
